# Recent advances in migraine therapy

**DOI:** 10.1186/s40064-016-2211-8

**Published:** 2016-05-17

**Authors:** Fabio Antonaci, Natascia Ghiotto, Shizheng Wu, Ennio Pucci, Alfredo Costa

**Affiliations:** Headache Center, C. Mondino National Neurological Institute, Pavia, Italy; Department of Brain and Behavioral Sciences, University of Pavia, Pavia, Italy; China Qinghai Provincial People’s Hospital, Xining, China

**Keywords:** Migraine, Acute treatment, Preventive treatment

## Abstract

Migraine is a common and highly disabling neurological disorder associated with a high socioeconomic burden. Effective migraine management depends on adequate patient education: to avoid unrealistic expectations, the condition must be carefully explained to the patient soon as it is diagnosed. The range of available acute treatments has increased over time. At present, abortive migraine therapy can be classed as specific (ergot derivatives and triptans) or non-specific (analgesics and non-steroidal anti-inflammatory drugs). Even though acute symptomatic therapy can be optimised, migraine continues to be a chronic and potentially progressive condition. In addition to the drugs officially approved for migraine prevention by international governmental regulatory agencies, numerous different agents are commonly used for this indication, showing various levels of evidence of efficacy and tolerability. Guidelines published in recent years, based on evidence-based medicine data on migraine prophylaxis, are a useful source of guidance, especially for primary care physicians and neurologists without specific expertise in headache medicine. Although the field of pharmacological migraine prevention has seen few advances in recent years, potential novel approaches are now being developed. This review looks at emerging pharmacological strategies for acute and preventive migraine treatment that are nearing or have already entered the clinical trial phase. Specifically, it discusses preclinical and clinical data on compounds acting on calcitonin gene-related peptide or its receptor, the serotonin 5-HT1F receptor, nitric oxide synthase, and acid-sensing ion channel blockers.

Migraine is a common and highly disabling primary headache form associated with a high socioeconomic burden and a generally high prevalence. Its prevalence is highest in the Americas, followed by Europe, whereas it is lower in Africa and Asia (Lipton et al. 2001).

Updated government statistics on the prevalence and burden of migraine and severe headache in the US indicate that migraine headaches affect around one in seven Americans annually (Burch et al. 2015), while summarised data from a review of headache and migraine in Europe showed the mean prevalence of current migraine among 170,000 adults to be 14.7 % (8 % in men and 17.6 % in women) (Lars and Colette 2010).

As regards the classification of migraine, the latest version of the International Headache Society’s International Classification of Headache Disorders, ICHD-III (beta) (Headache Classification Committee of the International Headache Society (IHS) 2013), contains a series of important changes, e.g. chronic migraine, previously classified as a complication of migraine, is now considered a migraine subcategory. Furthermore, the new version stipulates that patients with headache (tension-type-like and/or migraine-like) occurring on 15 or more days per month for more than 3 months, bearing the features of migraine headache on at least 8 days per month, must be classified as affected by chronic migraine (Headache Classification Committee of the International Headache Society (IHS) 2013). In this way the classification defines migraine according to attack frequency, and thus gives prominence to the most serious headache forms within the migraine spectrum.

Medication overuse is a major risk in chronic migraine; indeed, frequent use of some migraine medications can lead to the development of a disabling condition termed medication-overuse headache (MOH). In such cases, the overused drug needs to be withdrawn, after which the migraine will either revert to the episodic subtype or remain chronic, and must be re-diagnosed accordingly. Patients found to meet the criteria both for chronic migraine and for MOH should receive both diagnoses (Headache Classification Committee of the International Headache Society (IHS) 2013).

The initial diagnosis of migraine can be facilitated by the mnemonic POUND—pulsatile headache, 1-day duration (4–72 h), unilateral location, nausea or vomiting, and disabling intensity—, an evidence-based aid that summarises some of the main features of the condition.

A detailed medical history and neurological examination can then help confirm the diagnosis and rule out secondary conditions. Secondary headaches account for only a minority of headache diagnoses, but they can indicate the presence of severe, life-threatening conditions. Although prospective studies are lacking, there are various historical and physical features that, on the basis of extensive clinical experience, must be considered red flags for serious problems in this regard. It is important to look out for: older or younger age at onset (>50 years or <10 years); changes in presentation (increase in frequency and severity, headache that is worse than or different from the usual headache, precipitation of headache by Valsalva manoeuvres, progressive headache thunderclap headache; atypical or prolonged aura; first-ever onset of aura in women taking a combined oral contraceptive; concurrent systemic illness or systemic symptoms; abnormalities on neurological examination (Hainer and Matheson 2013; Dodick 1997; Orr et al. 2015).

Effective management of migraine depends on the provision of adequate patient education: to avoid unrealistic expectations, the condition must be carefully explained to the patient soon as it is diagnosed. Patient-compiled headache diaries can be very valuable tools for planning and evaluating treatment.

Migraine treatment traditionally includes both preventive therapy, aimed at reducing attack frequency and severity, and acute therapy, for aborting attacks.

Primarily, patients want to obtain faster onset of relief and more complete relief, without recurrences or adverse events. Accordingly, acute migraine treatment of a single attack seeks to provide rapid and definitive relief, with only minimal or no adverse events, and thus to promptly restore the patient’s ability to function (Silberstein et al. 2012a).

To achieve all this, it is necessary to follow several general rules: it is crucial to intervene early, when the pain is still mild, and to use adequate drug doses and appropriate routes of administration; antiemetic or prokinetic drugs should be co-administered to facilitate absorption of the primary drug; it is also important to take steps to avoid chronification of the headache and the development of MOH.

The two main approaches to migraine management are stratified care, which depends on the severity of the disease and assessment of other factors, and step care, in which patients are initially administered simple analgesics, but may subsequently need to progress to more powerful drugs (Lipton et al. 2000).

## Symptomatic therapy

The choice of acute treatment has increased over time. At present, the drugs that have shown efficacy in migraine attack therapy can be divided into: non-specific drugs (analgesics and non-steroidal anti-inflammatory drugs—NSAIDs) and specific drugs (ergot derivatives and triptans).

We here discuss the efficacy and safety of acute migraine drugs with reference to the recommendations contained in the European Federation of Neurological Societies guideline (Evers et al. 2009) and the guidelines of the American Academy of Neurology (Marmura et al. 2015).

On the basis of the quality of available studies, each acute migraine drug is assigned a level of evidence: level A, established as effective (or ineffective); level B, probably effective (or ineffective); level C, possibly effective (or ineffective); level U, evidence is conflicting or inadequate to support or refute the use of the medication(s).

## Non-specific drugs (analgesics and NSAIDs)

Despite growing use of the more recently introduced triptans, NSAIDs remain the most commonly used acute migraine treatments. A study examining migraine treatment patterns in the US population showed that nearly all (98 %) migraineurs used acute treatments, with 49 % using over-the-counter (OTC) medication only, 20 % using prescription medication only, and 29 % using both. The OTC medications included aspirin, other NSAIDs, paracetamol (acetaminophen), and paracetamol with caffeine (Diamond et al. 2007; Lipton et al. 2007). Large studies in France and Germany gave similar results (Lucas et al. 2006; Radtke and Neuhauser 2009).

Analgesics and NSAIDs are the drugs of first choice for mild or moderate migraine.

Phenazone 1000 mg (Göbel et al. 2004), metamizole 1000 mg (dipyrone) (Tulunay et al. 2004) and tolfenamic acid 200 mg have been shown to be effective acute migraine drugs (level B recommendation) (Evers et al. 2009). Acetaminophen alone is not recommended for moderate-to-severe migraine (level B recommendation) (Silberstein and US Headache Consortium 2000), although it can be used, at a dose of 1000 mg, for mild migraine (level A recommendation).

Acetylsalicylic acid (ASA), in doses of up to 1000 mg (Diener et al. 2004), ibuprofen (200–800 mg), diclofenac (50–100 mg) and naproxen sodium (500–1000 mg) (Evers et al. 2009; Suthisisang et al. 2010) are also effective (level A recommendation).

The fixed combination of ASA (250 mg), paracetamol (200 mg) and caffeine (50 mg) is more effective than these agents administered alone (Diener et al. 2005).

The oral combination of indomethacin, caffeine and prochlorperazine showed similar efficacy to oral sumatriptan, whereas its rectal formulation proved superior to oral sumatriptan (Sandrini et al. 2007). Patients using caffeine-containing drugs should be carefully monitored as they are more likely to develop rebound headache; caffeine can also contribute to the chronification process (Silbertsien and Strirpe 2014).

Antiemetics and prokinetics (such as domperidone 10 mg orally, metoclopramide 10 mg orally and prochlorperazine 3 mg orally) are indicated in addition to analgesics or NSAIDs for attacks accompanied by severe nausea and vomiting. Formulations containing NSAIDs (ASA or paracetamol) and metoclopramide are available in some countries, and paracetamol (1000 mg) in association with metoclopramide (10 mg) is superior to placebo (Evers et al. 2009).

Recent studies have shown that NSAIDs can also be administered by other routes. In a Class II study evaluating an intranasal formulation of ketorolac tromethamine 200 mg, containing 6 % lidocaine, for acute treatment (within 4 h of attack onset) of migraine with and without aura (Faffenrath et al. 2012) showed no significant difference in freedom from headache at 2 h between the ketorolac and placebo groups; however, ketorolac gave superior results on several secondary outcome measures, including lack of disability at 2 h and 2-h headache relief.

Intra-oral topical ketoprofen gel applied to a branch of the trigeminal nerve is being analysed in an ongoing randomised controlled trial (RCT) versus placebo (Behar 2011).

In a recent RCT (Lipton et al. 2010), a powder formulation of diclofenac potassium, to be dissolved in water, showed good efficacy in the treatment of moderate-to-severe migraine attacks.

Intravenous ibuprofen (800 mg) is undoubtedly a promising new NSAID formulation whose efficacy, versus placebo, as an acute migraine treatment is currently being investigated in an ongoing RCT (Latsko and Bradley 2015).

Etodolac is a new NSAID that showed good efficacy versus paracetamol in a recent RCT in 229 patients (Öztürk et al. 2013).

Several of the COX-2-specific inhibitors have also been studied as anti-migraine drugs. Rofecoxib and valdecoxib were both found to be effective but were subsequently withdrawn from the market due to concerns over an increased cardiovascular risk (Saper et al. 2006; Silberstein et al. 2004). Celecoxib 400 mg is still available and relieves acute migraine pain with the same efficacy as naproxen sodium 500 mg (Loo et al. 2007).

Several combinations of NSAIDs and triptans have been studied and the FDA has approved the combination of sumatriptan 85 mg and naproxen sodium 500 mg. This combination is superior to each of the agents used alone (Marmura et al. 2015).

In order to prevent the development of MOH, the intake of simple analgesics should be restricted to 15 days per month, and combined analgesics used on no more than 10 days per month.

Table 1 provides an overview of analgesics used in acute migraine treatment together with their levels of recommendation.

**Table 1:** Analgesics and non-steroidal anti-inflammatory drugs (NSAIDS) in migraine treatment

Substances	Dosages (mg)	Route(s) of administration	Maximum dosage per day (mg)	Level of recommendation
Acetylsalicylic acid (ASA)	325–650	Oral	4000	A
	300–600	Suppository		
	1000	Intravenous	4000	A
Ibuprofen	200–800 oral	Oral	3400	A
Naproxen sodium	250–750 oral	Oral	1250	A
Diclofenac	50–100 oral	Oral	150	
Paracetamol (acetaminophen)	325–1000	Oral	4000	A
	325–1000	Suppository	4000	
Metamizol (dipyrone)	250–1000	Oral	4000	B
	500–1000	Intravenous		
Phenazone	500–1000	Oral	4000	B
	500–1000	Suppository	4000	
Tolfenamic acid	200 mg	Oral	4000	B
ASA + acetaminophen + caffeine	250 + 200 + 50		2000 + 1600 + 400	B

The use of NSAIDs is contraindicated in patients with peptic ulcer and haemorrhagic diathesis. The long-term side effects (especially gastric symptoms) associated with ASA and other NSAIDs are well documented. However, in short-term trials ASA was generally well tolerated. NSAIDs have consistently shown lower overall adverse event rates (in particular, lower rates of nausea and vomiting) when compared with ergotamine.

## Specific anti-migraine drugs

### Ergot derivatives (ergotamine tartrate and dihydroergotamine)

The ergot derivatives exert an anti-migraine effect as a result of their agonist action on serotonin (5-HT) receptors, a mechanism of action shared by the triptans. Ergotamine, introduced in 1926, was the first drug used for acute migraine treatment. It remains a popular symptomatic migraine drug, probably because it is inexpensive and has a long duration of action. Ergotamine is used in the treatment of long-lasting attacks with tendency to headache recurrence (return of pain after initial treatment success). It is available in different formulations: oral tablet (0.5–2 mg), suppository (1–2 mg) and as a formulation for inhalation (maximum dosage 1.8 mg). In some countries it is used alone, although the suppository formulation usually contains a combination of ergotamine and caffeine.

The vasoconstrictor effect of ergotamine contraindicates its use in uncontrolled hypertension, coronary heart disease, peripheral vascular diseases, stroke, impaired hepatic or renal function, and pregnancy.

Because of their complex pharmacology and prolonged interaction with many other receptors (5-HT1A, 5-HT5, 5-HT2, 5-HT7, α-adrenoceptors and dopamine D2 receptors), which outlasts their long duration of action, ergot derivatives generate frequent and various adverse effects (most commonly nausea and vomiting, but also cramps, sleepiness and transient lower limb muscle pain). Ergotamine should not be taken within 6 h of triptans, and similarly triptans should not be administered within 24 h of ergotamine.

Dihydroergotamine (DHE) is usually better tolerated than ergotamine, but less effective due to its poor oral bioavailability. Intranasal DHE has better bioavailability (about 40 %), but a relatively slow onset of action. In two trials it was clearly shown to be inferior to intranasal and subcutaneous sumatriptan (Boureau et al. 2000; Touchon et al. 1996). Parenteral DHE, i.e. administered as a solution that can be injected intravenously or subcutaneously, is more effective in severe migraine attacks but produces more side effects (Colman et al. 2005).

A major problem with ergot derivatives is ergotamine-induced headache and rebound headache associated with frequent use. These problems can be limited by introducing a preventive therapy as the headache becomes more frequent. However, ergotamine can no longer be considered a drug of choice as it carries a high risk of overuse.

### Triptans

Introduced nearly 25 years ago, the triptans, a class of selective and highly effective 5-HT1B/1D receptor agonists, have now largely replaced the ergot derivatives.

The triptans are potent vasoconstrictors that seem to act on migraine by three main mechanisms: (1) intracranial extracerebral vasoconstriction, and inhibition of neurotransmitter release at both peripheral and central trigeminal nociceptive terminals, mainly via 5-HT1B/1D receptors (trigeminovascular afferents and trigeminal nucleus caudalis).

Sumatriptan, introduced in 1991, is the oldest drug in this class and still regarded as the gold standard (McCrory and Gray 2003; Winner et al. 2005), however, it has several drawbacks: low bioavailability, a short plasma half-life and low liposolubility. The six second-generation triptans (zolmitriptan, naratriptan, rizatriptan, almotriptan, eletriptan and frovatriptan), which entered the market more recently, show better pharmacokinetic properties (Table 2).

**Table 2:** The pharmacokinetic properties of the available triptans

Drug	Formulation	Dosage (mg)	Maximum 24 h dose (mg)	Time of peak levels	Elimination half life (H)	Bioavailability (%)
Sumatriptan	SI	6	6	12 min	2	97
	OT	50–100	200	2–3 h	2	15
	NS	20	40	1 h	2	17
Rizatriptan	ODT	10	30	1–2 h	2	14
Zolmitriptan	ODT	2.5	10	3 h	2.5–3	40–50
Almotriptan	OT	12.5	12.5	1.5–2 h	3.5	70
Eletriptan	OT	40	80	1.5–2 h	4	50
Naratriptan	OT	2.5	5	2–3 h	6	60–70
frovatriptan	OT	2.5	7.5	2–4 h	26	20–40

*SI* subcutaneous injection, *OT* oral tablet, *NS* nasal spray, *ODT* oral dispersible tablet, *min* minutes, *h* hour

The pharmacokinetic differences between the various triptans influence their use in clinical practice (Table 3).

**Table 3:** Use of the different triptans in clinical practice

Drug	Formulation	Use in clinical practice
Sumatriptan	SI	Rapid onset attack—if nausea/vomiting
	OT	
	NS	If nausea/vomiting
	S	If nausea/vomiting
Rizatriptan	ODT	Fast acting; if nausea/vomiting
	OT	
Zolmitriptan	ODT	If nausea/vomiting
	OT	
	NS	If nausea/vomiting
Almotriptan	OT	Previous adverse events
Eletriptan	OT	
Naratriptan	OT	Previous adverse events
		Long-lasting attacks
Frovatriptan	OT	Long-lasting attacks

*SI* subcutaneous injection, *OT* oral tablet, *NS* nasal spray, *ODT* oral dispersible tablet, *S* suppository

The efficacy of the triptans in migraine attack treatment has been extensively investigated in randomised, double-blind, placebo-controlled clinical trials, which have also attempted to define their optimal doses.

The measures of efficacy used in this context include: degree of pain relief, pain freedom at 2 h, sustained pain freedom (defined as pain-free at 2 h plus no use of rescue medication and no recurrence within 24 h), and sustained pain freedom associated with no adverse events.

The main literature findings are summarised below.

#### Sumatriptan

As shown by the results of systematic reviews and meta-analyses (Derry et al. 2012), the subcutaneous, oral and intranasal preparations of sumatriptan have shown evidence of efficacy in randomised, placebo-controlled trials of acute migraine therapies; furthermore, in a single placebo-controlled trial, the novel transdermal formulation was also found to be effective (Goldstein et al. 2012). Subcutaneous sumatriptan (6 mg) has the fastest onset of action and is more effective than oral sumatriptan (100 mg), but it is associated with more frequent adverse events. Oral sumatriptan is most effective at 100 mg, although the 50 mg dose may provide the best combination of efficacy and tolerability (Evers et al. 2009).

A sumatriptan transdermal patch was recently marketed; the dose delivered by the patch penetrates the skin using an electric gradient system (Schulman et al. 2010; Smith et al. 2011). Transdermal iontophoretic delivery of sumatriptan may offer migraine patients significant clinical benefits. Indeed, it is less likely to aggravate the gastrointestinal disturbances associated with migraine; furthermore, it is able to guarantee consistent, predictable delivery of desired drug levels over a 4-h period, and may avoid the atypical pain, pressure and other sensations commonly associated with current triptan formulations (Pierce et al. 2009).

A sumatriptan lingual spray, currently under development, has shown a good bioequivalence with oral sumatriptan 50 mg (Dilone et al. 2009).

#### Zolmitriptan

The efficacy of zolmitriptan as an acute migraine therapy has been shown by number of randomised, placebo-controlled trials, as well as by a meta-analysis. The optimal starting dose seemed to be 2.5 mg (Bird et al. 2014).

#### Naratriptan

According to least three randomised trials, naratriptan provides significantly better acute migraine relief than placebo does. In one of the studies, 2.5 mg was the most effective dose for producing headache relief at 4 h, showing an adverse event rate similar to that of placebo (Havanka et al. 2000; Stark et al. 2000).

#### Rizatriptan

A systematic review of seven randomised, placebo-controlled studies involving 3528 patients (Winner et al. 2003) showed that rizatriptan is an effective acute migraine treatment. Rizatriptan (both 5 and 10 mg), compared with placebo, provided a significant benefit in all five main efficacy outcomes (ranging from relief at 1–24 h) (Oldman et al. 2001). The 10 mg dose was the more effective; 5 mg is indicated if the patient is taking beta-blockers. Rizatriptan is the oral triptan with the fastest onset of efficacy (30 min) and the fastest time to peak plasma levels (60 min), but it also has the shortest plasma half-life (2–2.5 h).

#### Eletriptan

In a meta-analysis of six randomised, controlled trials of eletriptan involving 3224 patients, eletriptan at doses of 20, 40, and 80 mg performed significantly better than placebo for all the main outcomes (Pringsheim and Becker 2014). Pain relief was dose dependent, with 80 mg found to provide statistically significantly greater pain relief than 40 mg at both 2 and 24 h (Smith et al. 2001).

#### Frovatriptan

In randomised, double-blind trials the proportion of patients meeting the primary endpoint (a headache response at 2 h) was consistently found to be significantly higher among those taking frovatriptan as opposed to placebo. Three crossover trials compared early administration of frovatriptan 2.5 mg with almotriptan 12.5 mg, rizatriptan 10 mg and zolmitriptan 2.5 mg in patients with migraine. No significant between-group differences emerged in patient drug preference scores (primary endpoint) or in other endpoints, except for headache recurrence, which in two of the trials favoured frovatriptan (Sanford 2012). Frovatriptan was generally better tolerated than all four triptan comparators. In just one trial it failed to show the same efficacy as sumatriptan. Administration of frovatriptan early on in an attack, while the pain is still mild, was found to be more effective than late administration. In clinical trials frovatriptan appeared to show a more sustained treatment effect, having the longest plasma half-life (26 h) and the lowest recurrence rate at 24 h (7–25 %). Furthermore, it was better tolerated than the comparators.

Frovatriptan is recommended by a number of national guidelines for the perimenstrual prevention of menstrual attacks in women with pure menstrual migraine (MM) or menstrually related migraine (MacGregor 2014).

#### Almotriptan

Large RCTs have shown that almotriptan 12.5 mg provides optimal pain relief and tolerability. Almotriptan effectively improved pain relief at 2 h, reduced migraine-associated symptoms, and showed low recurrence rates. These findings were replicated in patient subgroups, such as adolescents and women with MM. These trials also showed that outcomes were better if patients took almotriptan early, when the pain was still mild. The clinical evidence collected and the comparisons made in the course of a decade of use have demonstrated that almotriptan is one of the more effective and fast-acting triptans available, showing a placebo-like tolerability profile (Antonaci et al. 2010; Pascual et al. 2010). The possibility of administering almotriptan via iontophoretic transdermal patch has also been raised (Calatayud-Pascual et al. 2011).

All the triptans are indicated (level A recommendation) for the acute treatment of moderate-to-severe migraine and mild-to-moderate migraine responding poorly to NSAIDs or combinations of analgesics, and mild-to-moderate migraine in patients with contraindications, intolerance or hypersensitivity to NSAIDs. The triptans are effective in about 60 % of NSAID non-responders.

Although most triptans are effective when administered orally, they are available in various formulations (tablets, oral dispersible tablets, nasal spray, subcutaneous injection, suppositories). Sumatriptan is the most flexible triptan in this sense, being available in subcutaneous injection, oral tablet, nasal spray, oral dispersible tablet and suppository forms.

The choice of triptan and formulation depends on the individual patient’s characteristics, preferences and headache features, as well as on convenience and cost considerations. The single patient’s response to a triptan cannot be predicted. The triptans are most beneficial if they are taken at the very onset of headache (Belvís et al. 2014).

Headache recurrence can occur in 15–40 % of patients taking oral triptans.

The efficacy of the triptans, in cases in which switching between triptans has failed to produce the desired effectiveness, could be improved by adding an NSAID.

All the triptans show similar side effects, but these are more marked when they are administered subcutaneously. Side effects may be mitigated by switching to a different triptan or choosing another route of administration. The most common triptan side effects are known as ‘triptan sensations’ and they include paraesthesias, flushing, tingling, neck pain and mild transient chest pressure. Although rare, cardiovascular complications have also been reported as the triptans can activate the (5-HT2A) receptor in peripheral arteries. The incidence of cardiac arrhythmias, myocardial infarct and stroke is about 1 in 1,000,000 patients under triptan treatment.

No association between triptan use and cardiovascular risk emerged from a systematic review of observational studies (Dodick et al. 2004). Similarly, a cohort study that included 13,664 migraine patients who were receiving a triptan found no association between triptan prescription and stroke, other cardiovascular events or death (Hall et al. 2004). However, in this cohort, triptans were prescribed to patients at less risk of these events. To date, few comparative observational studies have investigated the cardiovascular safety of migraine-specific drugs in clinical practice. On the basis of the available evidence, the triptans do not seem to be associated with major cardiovascular safety issues, although the evidence on stroke risk is conflicting. However, it has been suggested that if an increase of the absolute stroke risk in recently exposed patients does exist, it must be small (Roberto et al. 2015).

Acute migraine treatment with triptans is contraindicated in various circumstances, listed in Table 4. It should be noted that when treating cases of migraine with typical aura, triptans should be taken when the pain begins and not in the aura phase (Table 4).

**Table 4:** Contraindications of triptans

Avoid triptans in the presence of:
untreated arterial hypertension
coronary heart disease
Raynaud’s disease
a history of ischaemic stroke
pregnancy, breastfeeding
severe liver or kidney failure
age ≥ 65 years
basilar or hemiplegic migraine

The administration of other vasoconstrictors, such as ergotamine and its derivatives, and also of other triptans, is contraindicated within 24 h of taking a triptan.

Worldwide, sumatriptan and zolmitriptan have been approved only as nasal formulations for the treatment of migraine attacks in children older than 12 years. In addition, in the US, the use of oral almotriptan is approved in children older than 12 years, and oral rizatriptan 5 mg in children older than 6 years.

The development of MOH is a risk with all the triptans, which, therefore, must not be taken on more than 10 days per month (Headache Classification Committee of the International Headache Society (IHS) 2013). Literature data suggest that the mean time to onset of MOH is 1.7 years for triptan users, 2.7 years for patients taking ergots, and 4.8 years for those using analgesics (Limmroth et al. 2002).

## General principles of preventive treatment

Successful management of migraine rests on an effective alliance between the doctor and the patient, and on effective patient education: the diagnosis must be carefully explained to the patient from the outset and it must be properly understood. In this way, realistic expectations can be set. Patient-compiled headache diaries can be very valuable tools for planning and evaluating treatment. The availability of a carefully kept record of days with headache, pain severity, medication use and response, as well as obvious triggers (e.g., menstruation), can be vital in determining the need for preventive strategies and in evaluating therapeutic outcomes (Antonaci et al. 2011). Disability scales, such as the Migraine Disability Assessment Score (MIDAS) (Stewart et al. 1999), are important instruments for evaluating the impact of headache on a patient’s daily life. The Headache Under-Response to Treatment questionnaire allows even non-expert clinicians to measure the effectiveness of a headache treatment (Westergaard et al. 2013).

Migraine is a highly heterogeneous condition (Nappi et al. 2000) and its treatment should be tailored to the individual patient. After explaining the diagnosis, the physician should encourage the patient to actively participate in assessing how his/her lifestyle and behaviours (i.e. diet, sleep, exercise, avoidance of migraine-triggering factors) may contribute to his/her condition; patients should also be encouraged to explore non-pharmacological approaches whose effectiveness in migraine prevention is well documented, such as biofeedback, relaxation therapy and cognitive behavioural therapies.

There is some recent evidence of the effectiveness of behavioural interventions to supplement drug therapy with relaxation and cognitive behavioural therapy (CBT). Relaxation training (deep breathing) was easily adopted and often used post intervention. The CBT components were mainly viewed positively but regarded as more challenging to learn and implement in migraine (Morgan et al. 2015).

Although there are no sound and established criteria for choosing one prophylactic drug over another, there are various aspects and considerations that should be taken into account in order to weigh up the risk-benefits ratio: comorbidities, drug interactions, contraception, possible side effects, patient expectations, cost (Table 5).

**Table 5:** Crucial points in migraine therapy management

Educational programme	Avoid migraine-triggering factors
Monitoring attacks	Diary
Disability and outcome evaluation	Self-administered questionnaire
Non-pharmacological strategies	Relaxation therapy
	Biofeedback
	Cognitive behavioural techniques (CBT)
Pharmacological therapy	Comorbidities
	Side effects
	Drug interactions
	Patient expectations
	Cost

The US Headache Consortium defined the following goals for preventive treatment: (1) to decrease attack frequency by 50 % and decrease attack intensity and duration; (2) to improve responsiveness to acute therapy; (3) to improve function and decrease disability; and (4) to prevent the occurrence of MOH and chronic daily headache (Silberstein et al. 2012a). In general, a preventive treatment may be considered warranted (Lipton et al. 2007) when migraine attacks occur on 4 or 5 days per month with normal functioning, or when a patient experiences 2–3 migraine days per month with some impairment or disability.

The initiation of a preventive migraine therapy must be preceded by a discussion with the patient, who must be made aware of the treatment plan, the risk of side effects, and the duration of the proposed treatment. Drugs should be started at a low dose and slowly up-titrated until a therapeutic effect is seen, the maximum dose is achieved, or side effects become intolerable. All drugs should be trialled at an adequate dose for at least 6–8 weeks before being deemed ineffective (Freitag and Shumate 2014). Most patients need to be treated for at least 6 months in order to control their migraines; thereafter the drug can be gradually withdrawn.

Preventive treatment will not stop all attacks (two-thirds of patients can expect a 50 % reduction in headache frequency and patients need to understand that they will still need acute medication) (Table 6).

**Table 6:** Take-home message for optimal prevention

Involve patients in their care to improve adherence
Consider comorbidities and, when possible, choose a single medication to treat multiple comorbid disorders
When the patient is a woman of childbearing age, discuss contraception and the potential risk of medication use during pregnancy
Start at a low dose
Give each preventive medication at an adequate dose and for an adequate time (6–8 months)
Avoid interfering, contraindicated or overused medications
Re-evaluate the therapy; follow-up is important

## Pharmacological migraine prevention

Various drug classes are used for migraine prophylaxis, and several clinical practice guidelines have been published in the US and in Europe. Despite some differences, these are based on the same general principles.

The drugs of first choice in the US are three beta blockers (propranolol, timolol and metoprolol), and two antiepileptic drugs (divalproex sodium and topiramate). OnabotulinumtoxinA has also been approved for use in the treatment of chronic migraine, but not episodic migraine.

As regards the beta adrenergic blockers, propranolol (at doses of 80–240 mg per day), timolol (10–15 mg twice a day) and metoprolol (50–200 mg per day) are effective in migraine prophylaxis (Silberstein et al. 2012a). They are associated with a range of possible side effects: fatigue, sleep disorders, depression, decreased exercise tolerance and, less commonly, orthostatic hypotension, significant bradycardia and impotence.

Contraindications to treatment with these drugs include: congestive heart failure, asthma and insulin-dependent diabetes. Since metoprolol at doses of less than 200 mg per day preserves its beta-1 selectivity, it may be suitable for some patients with asthma and diabetes mellitus.

Several studies have shown the non-specific calcium channel blocker flunarizine to be effective in migraine prophylaxis at doses of 5–10 mg per day. Weight gain and depression are possible side effects. This drug is not available in the USA, but it is commonly used in some European countries, in Canada and in South America (Evers et al. 2009; Pringsheim et al. 2012).

As already indicated, antiepileptic drugs are also used in the prophylactic treatment of migraine. Divalproex sodium at doses of 500–1000 mg per day shows good efficacy and tolerability; its possible side effects are weight gain, hair loss and tremor. Valproate is a severely teratogenic drug and not be used as first-line migraine prophylaxis in women of child-bearing age. Topiramate is effective at doses of 100 mg per day, and has been studied in chronic migraine (Lipton and Silberstein 2015) versus placebo. Its possible side effects include behavioural or cognitive disturbances, impaired vision due to increased intraocular pressure, weight loss, renal stone formation, and a tingling or prickling sensation in the hands and feet. Most adverse events are minimised by titrating the dose by 25 mg per week to reach the target dose. Topiramate has been associated with cleft palate in newborns whose mothers used the drug during the first trimester of pregnancy and therefore cannot be considered the first-choice drug in women of child-bearing age.

OnabotulinumtoxinA is the only agent specifically approved for the prevention of chronic migraine (Silberstein et al. 2013). It is extremely well tolerated. A comparative trial versus topiramate in chronic migraine (Cady et al. 2011) showed similar results, although fewer adverse events were recorded in the onabotulinumtoxinA group.

Optimal use of injectable onabotulinumtoxinA depends on accurate delivery of the toxin to the target, as well as use of the correct dose and adherence to the recommended number of injections per site and frequency of injections (Ashkenazi and Blumenfeld 2013). The drug is typically injected across 31 sites on the head and neck (injected muscles include the procerus, bilateral corrugators, frontalis, temporalis, occipitalis, cervical paraspinal, and superior trapezius muscles). The recommended dose is 155 units administered intramuscularly (5 units per site), with retreatment every 12 weeks (Pringsheim et al. 2012).

A recent doses comparison study with OnabotulinumtoxinA in a real-life clinical setting demonstrated the superior efficacy of OnabotulinumtoxinA 195 U compared to 155 U in CM patients with MOH during a 2-year treatment period with similar safety and tolerability profile (Negro et al. 2015a). In a later contribution (2-years treatment) with OnabotulinumtoxinA 155 U it has been confirmed the efficacy and safely in chronic migraine but also in patients affected with and medication overuse headache (Negro et al. 2015b).

Finally, since studies of treatment with antidepressants were small, this category of drugs was not assigned a recommendation level A. That said, amitriptyline (doses of 10–150 mg per day), fluoxetine (doses of 10–40 mg per day) and venlafaxine extended release (doses of 75–150 mg per day) seem to be effective (Evers et al. 2009).

## Treatment in children and adolescents

The efficacy and safety of acute or preventive medications in children and adolescents are poorly documented. It goes without saying that these are two populations in which non-pharmacological approaches should be preferred. However, when a pharmacological therapy is warranted in a child or adolescent, the general principles are the same as those outlined for adults (Termine et al. 2011).

### Symptomatic therapy

A systematic review found acetaminophen (dose: 15 mg/kg) and ibuprofen (7.5–10 mg/kg) to be safe and effective in children (Lewis et al. 2004).

Worldwide, sumatriptan and zolmitriptan has been approved only as nasal formulations for the treatment of migraine attacks in children older than 12 years. In addition, in the US, the use of oral almotriptan is approved in this group, while oral rizatriptan 5 mg is approved for use in children older than 6 years of age (Evers 2013).

### Preventive therapy

Only two agents have been shown in multiple controlled trials to be effective in migraine prophylaxis in this age group: flunarizine 5 mg per day, which can be administered in the early evening to overcome the problem of daytime sleepiness, and topiramate. Topiramate effectively reduced headache frequency, severity and duration. The most common side effects reported were weight loss, cognitive problems and sensory disturbances (Lewis et al. 2004).

## Novel acute and preventive treatments

Recent years have seen the emergence of novel acute and preventive migraine treatments that target different neural mechanisms and/or act via innovative delivery systems (Rapoport 2011).

### Calcitonin gene-related peptide antagonists (gepants)

Calcitonin gene-related peptide (CGRP) is a neuropeptide implicated in the pathophysiology of migraine. Recent evidence suggests that the brainstem is the site of the key dysfunctions underlying migraine. It is thought that cortical changes, brainstem changes, or both, lead to activation of the trigeminal system. When this occurs, neuropeptides such as CGRP are released from trigeminal nerve endings in the meninges, inducing vasodilatation and triggering sensory trigeminal pain.

The CGRP blockers or receptor antagonists, also known as the “gepants” or monoclonal antibodies, are an interesting group of molecules which are emerging as possible new treatments for migraine. Unlike conventional treatments, they do not have a vasoconstrictive effect (Tso and Goadsby 2014) and, contrary to the current preventive strategies, they are able to target specific migraine mechanisms. They also represent the most prolific class: multiple agents targeting either CGRP itself or its receptor are under development for both acute and preventive migraine treatment.

Considering that CGRP receptor antagonists (CGRP-RAs) are the first non-serotoninergic, migraine-specific drugs without a vasoconstrictor action, it has been suggested that they may be suitable for patients with vascular disease, such as coronary artery disease and peripheral vascular disease (Tso and Goadsby 2014).

Five distinct CGRP-RAs (telcagepant, MK-3207, olcagepant, BMS-927711, BI44370TA, NCT01613248) have shown proof of efficacy for the treatment of migraine, but in all cases the trials were discontinued for various reasons, including concerns over the risk of liver toxicity with frequent use.

Telcagepant has been studied as a preventive therapy in a parallel-group RCT in episodic migraine patients. Although this trial, too, was terminated early due to concerns over hepatotoxocity, the limited data produced pointed to a larger reduction in mean migraine/probable migraine days in the telcagepant groups versus placebo (Ho et al. 2014).

Recently anti-GCRP antibodies (ALD-403, LY-2951742, LBR-101) have been developed as a means of removing the excess CGRP that is released from perivascular trigeminal nerve endings. Monoclonal antibodies against CGRP receptor (e.g. AMG-334) have also been developed to block the receptor from signalling transmission. The development of monoclonal antibodies against CGRP and its receptor may be seen as an illustration of how translational research is becoming part of a new approach to migraine prevention (Bigal et al. 2015). Data from phase I and II studies show the efficacy of these molecules versus placebo in episodic and chronic migraine (de Hoon et al. 2013).

### Serotonin 5HT1F agonists (ditans)

Another interesting drug is lasmiditan (COL-144), a selective 5-HT1F agonist at the receptor that has shown good efficacy and tolerability as an acute treatment in two RCTs (intravenous and oral administration) (CoLucid Pharmaceuticals 2008). Activation of 5-HT1F receptors decreases the expression of c-Fos, a marker of neuronal activation, in the trigeminal nucleus caudalis, without having vascular effects. In a randomised, phase II trial lasmiditan performed better than placebo at the highest doses. The most common adverse event was dizziness, present in up to 38 % of patients (Farkkila et al. 2012).

### Glutamate receptor antagonists

Glutamate, which is released from neurons expressing 5-HT1B/1D/1F receptors in the trigeminal ganglia, is implicated in aspects of both migraine and migraine aura pathophysiology, including trigeminovascular activation, central sensitisation and cortical spreading depression (Andreou and Goadsby 2009; Ramadan 2003). There are three glutamate receptor subtypes: *N*-methyl-d-aspartate (NMDA), α-amino-3-hydroxy-5-methyl-4-isoazolepropionic acid (AMPA), and kainate.

Some glutamate antagonists (tezampanel, LY-293558, and ADX10059) have shown effectiveness versus placebo in the acute treatment of migraine without aura. ADX10059 was studied in a multicentre migraine prevention study that was terminated early following the emergence of a higher than expected rate of liver enzyme abnormalities (Chan and MaassenVanDenBrink 2014).

Since it is known that NMDA receptors are activated or inhibited by neuroactive compounds generated by tryptophan metabolism through the kynurenine pathway, recent findings showed serum levels of all kynurenine metabolites altered in patients with chronic migraine and cluster headache (Curto et al. 2015). Infact a reduction in kynurenic acid levels was shown in patients affected by chronic migraine as well as an increase in serum xanthurenic acid, such finding has been interpreted as a defensive mechanism aimed at reducing the extent of headache in migraine.

The increasing number of preclinical data that highlight the importance of the kynurenine pathway in the pathophysiology and treatment of chronic headache disorders may open new therapeutic perspectives (Curto et al. 2015).

### Orexin receptor antagonists (rexants)

Orexin A and B are neuropeptides that are synthesised in the hypothalamus and thought to play a role in nociception. Originally developed to treat insomnia, filorexant is a dual orexin receptor (1 and 2) antagonist that has completed a phase IIa trial for the prophylaxis of migraine (NCT01513291); however, it was found to be ineffective (Chabi et al. 2015).

### Nitric oxide synthase inhibitors

Nitric oxide synthase (NOS) produces nitric oxide, a gaseous vasodilator that may activate trigeminovascular fibres, triggering the release of CGRP. Neuronal NOS and inducible NOS inhibitor were not found to be effective for acute or preventive migraine treatment (Palmer et al. 2009; Hoivik et al. 2010).

### Neuromodulation

Occipital nerve stimulation (ONS) with implanted leads was studied as a possible treatment for chronic migraine in the multicentre ONSTIM trial (Silberstein et al. 2012b). However, no significant difference was found in the primary outcome (50 % reduction of pain severity) between the patients randomised to active stimulation (n = 105) and the sham stimulation patients (n = 52). In addition, the authors reported a high rate of device-related adverse events: lead migration occurred in 18.7 % of patients and persistent pain or numbness at the lead site in 21.5 % (Silberstein et al. 2012b). Different results were obtained in recent contribution with paresthesia-free cervical 10 kHz spinal cord stimulation at 24 weeks of treatment of 17 patients suffering from resistent chronic migraine. Half of the subjects reported a >30 % reduction in headache days, 36 % reported a reduction greater than 50 %, and eight subjects reverted to an episodic migraine pattern. Medication intake reduced significantly, and four subjects discontinued triptans use at 6-month follow-up. The significant reduction in the number of headache days in the studied population at 6 months, the relative high number of ‘responders’ and the substantial decrease in the headache-specific questionnaires scores compares favourably to results seen in studies with ONS (Arcioni et al. 2016).

Interest thus turned to non-invasive neurostimulation devices. In this context, however, efficacy studies versus sham are problematical as the stimulation may produce paresthesias or pain.

Transcranial magnetic stimulation (TMS) is another neuromodulation technique. TMS uses a fluctuating magnetic field to induce, in the underlying cortex, an electrical current that is thought to disrupt cortical spreading depression (Tso and Goadsby 2014).

### Neurostimulation

*Cefaly* is a device with supraorbital transcutaneous stimulation properties that shows efficacy in migraine prevention (Arcioni et al. 2016). It is safe and well tolerated: in a survey of 2313 users, only 4.3 % reported minor adverse events and even fewer (2 %) had to discontinue its use (Magis et al. 2013). Positive results have been confirmed also in patients experiencing a low frequency of attacks, showing significant improvements in multiple migraine severity parameters following a brief period of high frequency tSNS. Therefore, tSNS may be considered a valid option for the preventive treatment of migraine attacks in patients who cannot or are not willing to take daily medications, or in whom low migraine frequency and/or intensity would not require pharmacological preventive therapies (Russo et al. 2015).

### Single-pulse transcranial magnetic stimulation

The *Cerena Transcranial Magnetic Stimulator* is a handheld device that applies single pulses of TMS to the back of the head. The device is indicated for the acute treatment of migraine with aura and indeed received FDA approval for this indication in December 2013. However, due to its large size it is not available to patients. A similar, but smaller device, the Spring TMS (manufactured by eNeura) has also been approved by the FDA for use in patients with migraine with aura. The device is for adults (patients aged ≥18 years) and it should not be used for more than one treatment in 24 h. The device must not be used by patients who have magnetic metals in their head, neck or upper body or by people with pacemakers, deep brain stimulators or other types of implanted device. Patients with suspected or diagnosed epilepsy or a personal or family history of seizures should also avoid this treatment.

*GammaCore* is a handheld device used for the acute and preventive treatment of migraine. By generating an electrical signal it delivers transcutaneous vagal nerve stimulation (VNS). A conductive gel is applied on the stimulation surfaces of the device and it is held against the neck. Each dose takes approximately two minutes to administer. VNS can suppress high glutamate levels in the trigeminal nucleus caudalis, and this may be the pain-blocking mechanism of non-invasive VNS (Oshinsky et al. 2014; Ambrosini et al. 2015). In a recent contribution a clinically meaningful response was observed after 3 months of prophylactic nVNS therapy in treatment-refractory migraine (both episodic and chronic) population. VNS was associated with significant reductions in pain intensity and number of headache days per month (Kinfe et al. 2015). Similar positive results were obtained in the acute treatment of high frequency episodic and chronic migraine that may help to reduce medication overuse and medication-associated adverse events (Barbanti et al. 2015).

Besides, transcutaneous stimulation of the auricular branch of the vagal nerve (t-VNS) has been used in the treatment of chronic migraine (Straube et al. 2015). A monocentric, randomized, controlled, double-blind study was conducted showing that the procedure was safe and effective. The mean reduction of headache days after 12 weeks of treatment exceeded that reported for other nerve stimulating procedures.

### Sphenopalatine ganglion stimulation

In view of the reported preventive effect of sphenopalatine ganglion stimulation in cluster headache, trials are now underway to explore the efficacy of this method as a possible preventive treatment of chronic migraine (Tso and Goadsby 2014; Khan et al. 2014).

## Conclusion

In summary, appropriate and patient-tailored migraine treatments can be identified only by evaluating five key aspects, according to the following decision-making pathway:

1. the level of attack frequency and disability (six or more headache days per month, more than four headache days with moderate disability, more than three headache days with impairment);
2. the suitability of acute therapy in the single patient (i.e. whether it is contraindicated, whether it produces adverse events, and whether or not it produces adequate relief);
3. the possible inefficacy of non-pharmacological therapy, or the patient’s unwillingness to use such approaches;
4. the presence of specific migraine forms (basilar, hemiplegic and prolonged aura migraine);
5. the presence of medical or psychiatric comorbidities.

In this way, the numerous variables that can modify the final approach (e.g. individual response, tolerability, patient preference and clinical aspects) are taken into account and concomitant medical or psychological conditions can be addressed and treated. It is also important to introduce measures aimed at reducing the biological tendency to headaches, but also to ensure that the patient has realistic expectations as regards what can be achieved. It is particularly important to identify and adequately treat patients affected by chronic migraine, given that this is a highly disabling but treatable clincal entity.

Since the therapeutic choice in migraine is quite elderly, pending the development of new acute and preventive options for migraine, our existing therapeutic armamentarium offers several options to be explored by clinicians in their efforts, working together with patients, to tackle the disability associated with migraine and improve the lives of migraine sufferers (Giamberardino and Martelletti 2015; Sabato et al. 2015; Martelletti 2015).

## References

[CR1] Ambrosini A, C D’Alessio C, Magis D, Schoenen J (2015) Targeting pericranial nerve branches to treat migraine: current approaches and perspectives. Cephalalgia. pii: 0333102415573511. (**Epub ahead of print**) 10.1177/0333102415573511 25736180

[CR2] Andreou AP, Goadsby PJ (2009). Therapeutic potential of novel glutamate receptor antagonists in migraine. Expert Opin Investig Drugs.

[CR3] Antonaci F, De Cillis I, Cuzzoni MG, Allena M (2010). Almotriptan for the treatment of acute migraine: a review of early intervention trials. Expert Rev Neurother.

[CR4] Antonaci F, Ghiotto N, Evangelista M (2011). An overview of the treatment options for acute migraine. Eur Neurol Rev.

[CR5] Arcioni R, Palmisani S, Mercieri M, Vano V, Tigano S, Smith T, Fiore MR, Al-Kaisy A, Martelletti P (2016). Cervical 10 kHz spinal cord stimulation in the management of chronic, medically refractory migraine: a prospective, open-label, exploratory study. Eur J Pain.

[CR6] Ashkenazi A, Blumenfeld A (2013). OnabotulinumtoxinA for the treatment of headache. Headache.

[CR7] Barbanti P, Grazzi L, Egeo G, Padovan AM, Liebler E, Bussone G (2015). Non-invasive vagus nerve stimulation for acute treatment of high-frequency and chronic migraine: an open-label study. J Headache Pain.

[CR8] Behar C (2011) A randomized double blind, placebo controlled phase III trial to determine the efficacy and safety of topical intra-oral ketoprofen for the treatment of acute migraine. In: ClinicalTrials.Gov. A service of the U.S. National Institutes of Health [online]. http://clinicaltrials.gov/. Accessed 11 Oct 2013

[CR9] Belvís R, Mas N, Aceituno A (2014). Migraine attack treatment: a tailor-made suit, not one size fits all. Recent Pat CNS Drug Discov.

[CR10] Bigal ME, Walter S, Rapoport AM (2015). Therapeutic antibodies against CGRP or its receptor. Br J Clin Pharmacol.

[CR11] Bird S, Derry S, Moore RA (2014). Zolmitriptan for acute migraine attacks in adults. Cochrane Database Syst Rev.

[CR12] Boureau F, Kappos L, Schoenen J (2000). A clinical comparison of sumatriptan nasal spray and dihydroergotamine nasal spray in the acute treatment of migraine. Int J Clin Pract.

[CR13] Burch RC, Loder S, Loder E, Smitherman TA (2015). The prevalence and burden of migraine and severe headache in the United States: updated statistics from government health surveillance studies. Headache.

[CR14] Cady RK, Schreiber CP, Porter JA, Blumenfeld AM, Farmer KU (2011). A multi-center double-blind pilot comparison of onabotulinum-toxinA and topiramate for the prophylactic treatment of chronic migraine. Headache.

[CR15] Calatayud-Pascual MA, Balaguer-Fernández C, Serna-Jiménez CE, Del Rio-Sancho S, Femenía-Font A, Merino V (2011). Effect of iontophoresis on in vitro transdermal absorption of almotriptan. Int J Pharm.

[CR16] Chabi A, Zhang Y, Jackson S, Cady R, Lines C, Herring WJ, Connor KM, Michelson D (2015). Randomized controlled trial of the orexin receptor antagonist filorexant for migraine prophylaxis. Cephalalgia.

[CR17] Chan K, Maassen VanDenBrink A (2014). Glutamate receptor antagonists in the management of migraine. Drugs.

[CR18] Colman I, Brown MD, Innes GD (2005). Parenteral dihydroergotamine for acute migraine headache: a systematic review of the literature. Ann Emerg Med.

[CR19] CoLucid Pharmaceuticals (2008) Clinical trial number NCT00384774 for “A Placebo-Controlled Adaptive Treatment Assignment Study of Intravenous COL-144 in the Acute Treatment of Migraine” at ClinicalTrials.gov Jump up. Clinical trial number NCT00883051 for “Dose-ranging Study of Oral COL-144 in Acute Migraine Treatment” at ClinicalTrials.gov

[CR20] Curto M, Lionetto L, Negro A, Capi M, Perugino F, Fazio F, Giamberardino MA, Simmaco M, Nicoletti F, Martelletti P (2015). Altered serum levels of kynurenine metabolites in patients affected by cluster headache. J Headache Pain.

[CR21] de Hoon J, Montieth D, Vermeersch S (2013). Safety, pharmacokinetics, and pharmacodynamics of LY2951742: a monoclonal antibody targeting CGRP. Cephalalgia Int J Headache.

[CR22] Derry CJ, Derry S, Moore RA (2012). Sumatriptan (oral route of administration) for acute migraine attacks in adults. Cochrane Database Syst Rev.

[CR23] Diamond S, Bigal ME, Silberstein S, Loder E, Reed M, Lipton RB (2007). Patterns of diagnosis and acute and preventive treatment for migraine in the United States: results from the American Migraine Prevalence and Prevention study. Headache.

[CR24] Diener HC, Bussone G, de Liano H, Eikermann A, Englert R, Floeter T (2004). Placebo-controlled comparison of effervescent acetylsalicylic acid, sumatriptan and ibuprofen in the treatment of migraine attacks. Cephalalgia.

[CR25] Diener HC, Pfaffenrath V, Pageler L, Peil H, Aicher B (2005). The fixed combination of acetylsalicylic acid, paracetamol and caffeine is more effective than single substances and dual combination for the treatment of headache: a multicenter, randomized, double-blind, single-dose, placebo-controlled parallel group study. Cephalalgia.

[CR26] Dilone E, Bergstrom D, Cabana B, Nedumpara M, Fox AW, Sumatriptan Lingual Spray Study Group (2009). Rapid oral transmucosal absorption of sumatriptan, and pharmacodynamics in acute migraine. Headache.

[CR27] Dodick D (1997). Headache as a symptom of ominous disease. What are the warning signals?. Postgrad Med.

[CR28] Dodick DW, Martin VT, Smith T, Silberstein S (2004). Cardiovascular tolerability and safety of triptans: a review of clinical data. Headache.

[CR29] Evers S (2013). The efficacy of triptans in childhood and adolescence migraine. Curr Pain Headache Rep.

[CR30] Evers S, Afra J, Frese A, Goadsby PJ, Linde M, May A, Sándor PS, European Federation of Neurological Societies (2009). EFNS guideline on the drug treatment of migraine–revised report of an EFNS task force. Eur J Neurol.

[CR31] Faffenrath V, Fenzl E, Bregman D, Färkkila M (2012). Intranasal ketorolac tromethamine (SPRIX(R)) containing 6% of lidocaine (ROX-828) for acute treatment of migraine: safety and efficacy data from a phase II clinical trial. Cephalalgia.

[CR32] Farkkila M, Diener HC, Geraud G (2012). Efficacy and tolerability of lasmiditan, an oral 5-HT(1F) receptor agonist, for the acute treatment of migraine: a phase 2 randomised, placebo-controlled, parallel-group, doseranging study. Lancet Neurol.

[CR33] Freitag FG, Shumate D (2014). Current and investigational drugs for the prevention of migraine in adults and children. CNS Drugs.

[CR34] Giamberardino MA, Martelletti P (2015). Emerging drugs for migraine treatment. Expert Opin Emerg Drugs.

[CR35] Göbel H, Heinze A, Niederberger U, Witt T, Zumbroich V (2004). Efficacy of phenazone in the treatment of acute migraine attacks: a double-blind, placebo-controlled, randomized study. Cephalalgia.

[CR36] Goldstein J, Smith TR, Pugach N (2012). A sumatriptan iontophoretic transdermal system for the acute treatment of migraine. Headache.

[CR37] Hainer BL, Matheson EM (2013). Approach to acute headache in adults. Am Fam Phys.

[CR38] Hall GC, Brown MM, Mo J, MacRae KD (2004). Triptans in migraine: the risks of stroke, cardiovascular disease, and death in practice. Neurology.

[CR39] Havanka H, Dahlöf C, Pop PH (2000). Efficacy of naratriptan tablets in the acute treatment of migraine: a dose-ranging study. Naratriptan S2WB2004 Study Group. Clin Ther.

[CR40] Headache Classification Committee of the International Headache Society (IHS) (2013). The international classification of headache disorders, 3rd edition (beta version). Cephalalgia.

[CR41] Ho TW, Connor KM, Zhang Y, Pearlman E, Koppenhaver J, Fan X, Lines C, Edvinsson L, Goadsby PJ, Michelson D (2014). Randomized controlled trial of the CGRP receptor antagonist telcagepant for migraine prevention. Neurology.

[CR42] Hoivik HO, Laurijssens BE, Harnisch LO (2010). Lack of efficacy of the selective iNOS inhibitor GW274150 in prophylaxis of migraine headache. Cephalalgia Int J Headache.

[CR43] Khan S, Schoenen J, Ashina M (2014). Sphenopalatine ganglion neuromodulation in migraine: what is the rationale?. Cephalalgia.

[CR44] Kinfe TM, Pintea B, Muhammad S, Zaremba S, Roeske S, Simon BJ, Vatter H (2015). Cervical non-invasive vagus nerve stimulation (nVNS) for preventive and acute treatment of episodic and chronic migraine and migraine-associated sleep disturbance: a prospective observational cohort study. J Headache Pain.

[CR45] Lars JS, Colette A (2010). Prevalence of headache in Europe: a review for the Eurolight project. J Headache Pain.

[CR46] Latsko M, Bradley K (2015) A double-blind, placebo-controlled pilot study to collect and evaluate data on the use of intravenous ibuprofen in the treatment of an acute migraine attack. In: ClinicalTrials.Gov. A service of the U.S. National Institutes of Health [online]. http://clinicaltrials.gov/)

[CR47] Lewis D, Ashwal S, Hershey A, Hirtz D, Yonker M, Silberstein S (2004). Practice parameter: pharmacological treatment of migraine headache in children and adolescents: report of the American Academy of Neurology Quality Standards Subcommittee and the Practice Committee of the Child Neurology Society. Neurology.

[CR48] Limmroth V, Katsarava Z, Fritsche G, Przywara S, Diener HC (2002). Features of medication overuse headache following overuse of different acute headache drugs. Neurology.

[CR49] Lipton RB, Silberstein SD (2015). Episodic and chronic migraine headache: breaking down barriers to optimal treatment and prevention. Headache.

[CR50] Lipton RB, Stewart WF, Stone AM, Láinez MJA, Sawyer JPC (2000). Stratified care vs step care strategies for migraine. The Disability in Strategies of Care (DISC) Study: a randomized trial. JAMA.

[CR51] Lipton RB, Stewart WF, Diamond S, Diamond ML, Reed M (2001). Prevalence and burden of migraine in the United States: data from the American Migraine Study II. Headache.

[CR52] Lipton RB, Bigal ME, Diamond M, Freitag F, Reed ML, Stewart WF (2007). AMPP Advisory Group Migraine prevalence, disease burden, and the need for preventive therapy. Neurology.

[CR53] Lipton RB, Grosberg B, Singer RP, Pearlman SH, Sorrentino JV, Quiring JN (2010). Efficacy and tolerability of a new powdered formulation of diclofenac potassium for oral solution for the acute treatment of migraine: results from the International Migraine Pain Assessment Clinical Trial (IMPACT). Cephalalgia.

[CR54] Loo CY, Tan HJ, Teh HS, Raymond AA (2007). Randomised, open label, controlled trial of celecoxib in the treatment of acute migraine. Singapore Med J.

[CR55] Lucas C, Géraud G, Valade D, Chautard MH, Lantéri-Minet M (2006). Recognition and therapeutic management of migraine in 2004, in France: results of FRAMIG 3, a French nationwide population-based survey. Headache.

[CR56] MacGregor EA (2014). A review of frovatriptan for the treatment of menstrual migraine. Int J Women’s Health.

[CR57] Magis D, Sava S, D’Elia TS (2013). Safety and patients’ satisfaction of transcutaneous supraorbital neurostimulation (tSNS) with the Cefaly^®^ device in headache treatment: a survey of 2,313 headache sufferers in the general population. J Headache Pain.

[CR58] Marmura MJ, Silberstein SD, Schwedt TJ (2015). The acute treatment of migraine in adults: the American headache society evidence assessment of migraine pharmacotherapies. Headache.

[CR59] Martelletti P (2015). The therapeutic armamentarium in migraine is quite elderly. Expert Opin Drug Metab Toxicol.

[CR60] McCrory DC, Gray RN (2003). Oral sumatriptan for acute migraine. Cochrane Database Syst Rev.

[CR61] Morgan M, Cousins S, Middleton L, Warriner-Gallyer G, Ridsdale L (2015). Patients’ experiences of a behavioural intervention for migraine headache: a qualitative study. J Headache Pain.

[CR62] Nappi G, Costa A, Tassorelli C, Santorelli FM (2000). Migraine as a complex disease: heterogeneity, comorbidity and genotype-phenotype interactions. Funct Neurol.

[CR63] Negro A, Curto M, Lionetto L, Martelletti P (2015). A two years open-label prospective study of OnabotulinumtoxinA 195 U in medication overuse headache: a real-world experience. J Headache Pain.

[CR64] Negro A, Curto M, Lionetto L, Crialesi D, Martelletti P (2015). OnabotulinumtoxinA 155 U in medication overuse headache: a two years prospective study. Springerplus.

[CR65] Oldman AD, Smith LA, McQuay HJ, Moore RA (2001) Rizatriptan for acute migraine. Cochrane Database Syst Rev (3):CD003221 10.1002/14651858.CD003221 11687054

[CR66] Orr SL, Aubé M, Becker WJ, Davenport WJ, Dilli E, Dodick D, Giammarco R, Gladstone J, Leroux E, Pim H, Dickinson G, Christie SN (2015). Canadian Headache Society systematic review and recommendations on the treatment of migraine pain in emergency setting. Cephalalgia.

[CR67] Oshinsky ML, Murphy AL, Hekierski H, Cooper M, Simon BJ (2014). Noninvasive vagus nerve stimulation as treatment for trigeminal allodynia. Pain.

[CR68] Öztürk V, Ertaş M, Baykan B, Sirin H, Özge A, Mig-Etol Study Group (2013) Efficacy and safety of 400 and 800 mg etodolac vs. 1,000 mg paracetamol in acute treatment of migraine: a randomized, double-blind, crossover, multicenter, phase III clinical trial. Pain Pract 13(3):191–197. doi:10.1111/j.1533-2500.2012.00572.x. 10.1111/j.1533-2500.2012.00572.x 22730906

[CR69] Palmer J, Guillard F, Laurijssens B (2009). A randomised, single-blind, placebo-controlled, adaptive clinical trial of GW274150, a selective iNOS inhibitor, in the treatment of acute migraine. Cephalalgia Int J Headache.

[CR70] Pascual J, Vila C, McGown CC (2010). Almotriptan: a review of 10 years’ clinical experience. Expert Rev Neurother.

[CR71] Pierce M, Marbury T, O’Neill C (2009). Zelrix: a novel transdermal formulation of sumatriptan. Headache.

[CR72] Pringsheim T, Becker WJ (2014). Triptans for symptomatic treatment of migraine headache. BMJ.

[CR73] Pringsheim T, Davenport W, Mackie G, Worthington I, Aubé M, Christie SN, Gladstone J, Becker WJ (2012). Canadian Headache Society guideline for migraine prophylaxis. Can J Neurol Sci.

[CR74] Radtke A, Neuhauser H (2009). Prevalence and burden of headache and migraine in Germany. Headache.

[CR75] Ramadan NM (2003). The link between glutamate and migraine. CNS Spectr.

[CR76] Rapoport A (2011). New frontiers in headache therapy. Neurol Sci.

[CR77] Roberto G, Raschi E, Piccinni C, Conti V, Vignatelli L, D’Alessandro R, De Ponti F, Poluzzi E (2015). Adverse cardiovascular events associated with triptans and ergotamines for treatment of migraine: systematic review of observational studies. Cephalalgia.

[CR78] Russo A, Tessitore A, Conte F, Marcuccio L, Giordano A, Tedeschi G (2015). Transcutaneous supraorbital neurostimulation in “de novo” patients with migraine without aura: the first Italian experience. J Headache Pain.

[CR79] Sabato D, Lionetto L, Martelletti P (2015). The therapeutic potential of novel anti-migraine acute therapies. Expert Opin Investig Drugs.

[CR80] Sandrini G, Cerbo R, Del Bene E, Ferrari A, Genco S, Grazioli I, Martelletti P, Nappi G, Pinessi L, Sarchielli P, Tamburro P, Uslenghi C, Zanchin G (2007). Efficacy of dosing and re-dosing of two oral fixed combinations of indomethacin, prochlorperazine and caffeine compared with oral sumatriptan in the acute treatment of multiple migraine attacks: a double-blind, double-dummy, randomised, parallel group, multicentre study. Int J Clin Pract.

[CR81] Sanford M (2012). Frovatriptan: a review of its use in the acute treatment of migraine. (CNS. Drugs.

[CR82] Saper J, Dahlof C, So Y (2006). Rofecoxib in acute treatment of migraine. A randomised controlled clinica trial. Headache.

[CR83] Schulman E, O ´Neill C, Pierce M, Griesser J, Angelov AS (2010) Efficacy of Zelrix, a novel iontophoretic transdermal sumatriptan patch, in the treatment of acute migraine in patients with nausea. Presented at the 62nd Annual Meeting of the American Academy of Neurology, April 10–17, 2010, Toronto

[CR84] Silberstein SD, US Headache Consortium (2000). Practice parameter: evidence-based guidelines for migraine headache (an evidence-based review). Report of the Quality Standards Subcommittee of the American Academy of Neurology. Neurology.

[CR85] Silberstein S, Tapper S, Brandes J (2004). Randomized, placebo-controlled trial of rofecoxib in the acute treatment of migraine. Neurology.

[CR86] Silberstein SD, Holland S, Freitag F, Dodick DW, Argoff C, Ashman E (2012). Evidence-based guideline update: pharmacologic treatment for episodic migraine prevention in adults: report of the quality standards subcommittee of the American Academy of Neurology and the American Headache Society. Neurology.

[CR87] Silberstein SD, Dodick DW, Saper J, Huh B, Slavin KV, Sharan A, Reed K, Narouze S, Mogilner A, Goldstein J, Trentman T, Vaisman J, Ordia J, Weber P, Deer T, Levy R, Diaz RL, Washburn SN, Mekhail N (2012). Safety and efficacy of peripheral nerve stimulation of the occipital nerves for the management of chronic migraine: results from a randomized, multicenter, double-blinded, controlled study. Cephalalgia.

[CR88] Silberstein SD, Blumenfeld AM, Cady RK, Turner IM, Lipton RB, Diener HC, Aurora SK, Sirimanne M, DeGryse RE, Turkel CC, Dodick DW (2013). OnabotulinumtoxinA for treatment of chronic migraine: PREEMPT 24-week pooled subgroup analysis of patients who had acute headache medication overuse at baseline. J Neurol Sci.

[CR89] Silbertsien SD, Strirpe J (2014). COX inhibitors for the treatment of migraine. Expert Opin Pharmacother.

[CR90] Smith LA, Oldman AD, McQuay HJ, Moore RA (2001) Eletriptan for acute migraine. Cochrane Database Syst Rev (3):CD003224 10.1002/14651858.CD003224 11687056

[CR91] Smith T, Pierce M, Griesser J (2011) An open-label study to evaluate the long-term safety of zelrixTM, a sumatriptan iontophoretic patch for the treatment of acute migraine. Presented at the 15th Congress of the International Headache Society, June 23–26, 2011, Berlin

[CR92] Stark S, Spierings EL, McNeal S (2000). Naratriptan efficacy in migraineurs who respond poorly to oral sumatriptan. Headache.

[CR93] Stewart WF, Lipton RB, Whyte J (1999). An international study to assess reliability of the migraine disability assessment (MIDAS) score. Neurology.

[CR94] Straube A, Ellrich J, Eren O, Blum B, Ruscheweyh R (2015). Treatment of chronic migraine with transcutaneous stimulation of the auricular branch of the vagal nerve (auricular t-VNS): a randomized, monocentric clinical trial. J Headache Pain.

[CR95] Suthisisang CC, Poolsup N, Suksomboon N, Lertpipopmetha V, Tepwitukgid B (2010). Meta-analysis of the efficacy and safety of naproxen sodium in the acute treatment of migraine. Headache.

[CR96] Termine C, Ozge A, Antonaci F, Natriashvili S, Guidetti V, Wöber-Bingöl C (2011). Overview of diagnosis and management of paediatric headache. Part II: therapeutic management. J Headache Pain.

[CR97] Touchon J, Bertin L, Pilgrim AJ (1996). A comparison of subcutaneous sumatriptan and dihydroergotamine nasal spray in the acute treatment of migraine. Neurology.

[CR98] Tso AR, Goadsby PJ (2014). New targets for migraine therapy. Curr Treat Options Neurol.

[CR99] Tulunay FC, Ergun H, Gulmez SE, Ozbenli T, Ozmenolu M, Boz C (2004). The efficacy and safety of dipyrone (Novalgin) tablets in the treatment of acute migraine attacks: a double-blind, cross-over, randomized, placebocontrolled, multi-center study. Functional Neurol.

[CR100] Westergaard ML, Steiner TJ, MacGregor EA, Antonaci F, Tassorelli C, Buse DC, Lipton RB, Jensen RH (2013). The Headache Under-Response to Treatment (HURT) Questionnaire: assessment of utility in headache specialist care. Cephalalgia.

[CR101] Winner P, Mannix LK, Putnam DG, McNeal S, Kwong J, O’Quinn S (2003). Richardson MS Pain-free results with sumatriptan taken at the first sign of migraine pain: 2 randomized, double-blind, placebo-controlled studies. Mayo Clin Proc.

[CR102] Winner P, Landy S, Richardson M, Ames M (2005). Early intervention in migraine with sumatriptan tablets 50 mg versus 100 mg: a pooled analysis of data from six clinical trials. Clin Ther.

